# Reply to Marques and Drexler, “Complex Scenario of Homotypic and Heterotypic Zika Virus Immune Enhancement”

**DOI:** 10.1128/mBio.02073-19

**Published:** 2019-09-03

**Authors:** Byoung-Shik Shim, Hyeryun Choe

**Affiliations:** a Department of Immunology and Microbiology, The Scripps Research Institute, Jupiter, Florida, USA; Vanderbilt University Medical Center

**Keywords:** Zika virus, antibody-dependent enhancement, homotypic

## REPLY

We generally agree with the thoughtful assessment of the role of ADE described in the letter of Marques and Drexler (1) and especially with the notion that the balance between ADE and protective immunity is variable across experimental conditions, among individuals, and within individuals over time. The authors also raised several concerns about the interpretation of our studies (2). First, they note that ADE observed in a murine model in which antibodies or immune plasmas are passively transferred is often not reproduced if immunity is induced by real virus infection. They are correct, but in the studies of real virus infection, usually insufficient time is allowed for protective responses to wane to the point where ADE can dominate. To determine the potential role of ADE, including homotypic ADE, one needs either several years to allow for sufficient attenuation of protective immunity or to show by passive infusion that low concentrations of serum (but not high concentrations) markedly worsen ZIKV-mediated disease. Therefore, negative ADE data also require a careful analysis of their specific contexts. Marques and Drexler also point out in their letter that “mothers of CZS cases had significantly higher, not lower, ZIKV-specific antibody titers than controls,” implying that ADE is not well correlated with reduced neutralizing antibody titers. There are many factors that contribute to this observation. One such factor is that higher antibody titers can imply higher maternal viral loads, increasing the likelihood of vertical transmission. In short, we do not think that these concerns undercut the key point of our paper: homotypic ZIKV ADE remains a serious concern in the absence of robust and persistent T-cell responses. We hope with the authors of the letter that our results will encourage more careful consideration of the balance between protective immunity and both hetero- and homotypic ADE in humans.
